# Pairwise Registration Algorithm for Large-Scale Planar Point Cloud Used in Flatness Measurement

**DOI:** 10.3390/s21144860

**Published:** 2021-07-16

**Authors:** Zichao Shu, Songxiao Cao, Qing Jiang, Zhipeng Xu, Jianbin Tang, Qiaojun Zhou

**Affiliations:** College of Metrology and Measurement Engineering, China Jiliang University, Hangzhou 310018, China; p20020854070@cjlu.edu.cn (Z.S.); jiangq2004@163.com (Q.J.); xuzhipeng@cjlu.edu.cn (Z.X.); tangjb@cjlu.edu.cn (J.T.); zqj@cjlu.edu.cn (Q.Z.)

**Keywords:** 3D point cloud registration, boundary extraction, boundary-based registration, flatness measurement

## Abstract

In this paper, an optimized three-dimensional (3D) pairwise point cloud registration algorithm is proposed, which is used for flatness measurement based on a laser profilometer. The objective is to achieve a fast and accurate six-degrees-of-freedom (6-DoF) pose estimation of a large-scale planar point cloud to ensure that the flatness measurement is precise. To that end, the proposed algorithm extracts the boundary of the point cloud to obtain more effective feature descriptors of the keypoints. Then, it eliminates the invalid keypoints by neighborhood evaluation to obtain the initial matching point pairs. Thereafter, clustering combined with the geometric consistency constraints of correspondences is conducted to realize coarse registration. Finally, the iterative closest point (ICP) algorithm is used to complete fine registration based on the boundary point cloud. The experimental results demonstrate that the proposed algorithm is superior to the current algorithms in terms of boundary extraction and registration performance.

## 1. Introduction

Flatness is an important component of geometric tolerance and has a wide range of testing applications in many fields, such as for precision machinery, military manufacturing, and electronics. The traditional flatness measurement scheme is limited by many factors, such as precision, detection speed, and equipment cost, which cannot meet the increasingly stringent detection requirements in production lines. Machine vision has become widely used for flatness measurement. Among various machine vision schemes, a laser profilometer [1,2] is suitable for fast and high-precision flatness measurement because of its excellent point cloud acquisition ability. However, to improve the repeatability of flatness measurement, the relative position of each sampling point must be fixed in repeated measurements, which is undoubtedly a difficult problem when the initial pose of the objects to be measured differs significantly. To solve the above problem, the rigid registration technology of a three-dimensional (3D) point cloud is used to achieve six degrees-of-freedom (6-DoF) [3,4] pose estimation of the object to be measured.

The iterative closest point (ICP) and its variants [5,6,7,8,9,10] are the most widely used point cloud registration methods due to their simplicity and good performance. However, the main problems in point cloud registration for flatness measurement are as follows: Because of its high computational complexity, the ICP algorithm cannot efficiently process large-scale point clouds obtained by a laser profilometer. In addition, the ICP method may suffer from the local optimum problem because it considers the closest point as the corresponding point [11], especially when the point cloud is not well initialized. Conversely, to overcome the local minimum problem, the coarse-to-fine registration strategy [12] is widely used. However, in the case of point clouds with curvature-invariant surfaces [13], the coarse registration precision is low, which adversely affects subsequent fine registration.

In this paper, an optimized 3D point cloud registration algorithm is proposed, which realizes the registration of large-scale planar point clouds in flatness measurement. We introduce an improved 3D point cloud boundary extraction method, which obtains approximate boundary points through coarse estimation. Then, it removes outliers and extracts accurate boundary points through fine estimation in the neighborhood of the approximate boundary points. Compared with the traditional boundary extraction method, the optimized method introduces the idea of a hierarchical strategy to avoid evaluating every point, thereby greatly improving the efficiency of calculation. Then, point cloud registration is acquired based on the boundary information. We calculate the feature descriptor of the boundary point cloud to overcome the problem of weak uniqueness of feature descriptors in curvature-invariant surfaces. We concurrently designed an algorithm to eliminate invalid keypoints and mismatches for the boundary point cloud to achieve fast and accurate coarse registration. Finally, the boundary point cloud is input to the ICP framework to accomplish fine registration, which significantly reduces the computational complexity and maintains accuracy. The experimental results show that the proposed algorithm can effectively extract the boundary of a 3D point cloud and obtain better registration accuracy and computation efficiency in several point clouds to ensure fast and accurate flatness measurement based on a laser profilometer. The main contributions of this study are as follows:

1. An improved 3D point cloud boundary extraction method is proposed. This method integrates a hierarchical strategy, an outlier filter, and a traditional boundary extraction algorithm to increase computational efficiency.
2. A novel boundary-based registration method is presented in this study. It achieves coarse-to-fine registration based on a boundary point cloud, which significantly improves the situation in which ICP is prone to fall into local minima and low calculation efficiency and has high registration precision.
3. We apply the proposed large-scale planar point cloud registration algorithm to the laser profilometer-based flatness measurement for greater efficiency and accuracy.

The remainder of this paper is organized as follows. Related work is described in Section 2. The proposed method is detailed in Section 3. The experimental results and analyses are presented in Section 4. Conclusions and perspectives are presented in Section 5.

## 2. Related Works

### 2.1. Boundary Extraction

Since the original point clouds obtained by the scanning device have a large number of points and contain a lot of curvature-consistent surfaces, boundary feature extraction is needed to improve data utilization. The point cloud boundary plays an important role in boundary-based registration. The efficient and accuracy of point cloud registration will be directly affected by the boundary extraction performance. A variety of boundary extraction methods have been developed. Wang et al. [14] introduced an approach to fusing boundary data extracted in a 2D image and 3D point cloud to obtain the 3D boundary characteristics with increased accuracy; however, the accuracy of identifying the target object from the background in the depth image may affect the final performance of the algorithm. Chen et al. [15] achieved point cloud boundary detection, which combined the *k*-nearest neighbors angle method [16,17] with the geometric distribution of point clouds. However, the applicability of the two-way nearest points search method is poor, and for the large-scale point clouds, using each point for *k*-nearest neighbors search will be time consuming. The improved Hough transform [18] was successfully applied to boundary extraction of point clouds, reducing the computational complexity, but the final result is susceptible to the performance of normal calculation. Chen et al. [19] used an improved *k*-d tree method to search the *k* neighbors in point cloud; then, they established the least-squares microcut plane by the sampling point and its *k*-nearest neighbors and projected them onto the plane, and the boundary points were extracted according to the judge criterion of field force and a sorting method based on the vector deflected angle and distance.

In general, existing boundary extraction algorithms can achieve high accuracy, but for large-scale point clouds, most of them take a long time because of the *k* neighbors searching and normal estimation for each point.

### 2.2. Coarse Registration

One of the core technologies for 3D point cloud data processing, 3D point cloud registration is widely used in many fields, such as 3D reconstruction [20], 3D recognition [21], and simultaneous localization and mapping (SLAM) [22]. For registration, it is critical to obtain a coordinate transformation matrix between a set of point clouds across several views into the same coordinate system using particular algorithms or statistical rules. The local registration algorithms easily fall into a local optimum when the initial pose of the point cloud sets is arbitrary. To solve this problem, a coarse-to-fine strategy is proposed, where the coarse registration can estimate a rough transformation matrix between two surfaces without strict requirements of the initial pose, and the fine registration refines the approximate transformation [23].

Handcrafted feature-based methods are widely used for coarse registration. These methods entail two significant steps: extracting geometric characteristics and identifying correspondences [24]. In step one, features such as fast point feature histogram (FPFH) [25], 3D scale-invariant feature transform (SIFT) [26], signature of histogram of orientation (SHOT) [27], rotation projection statistics (RoPS) [28], local feature statistic histogram (LFSH) [29], binary shape context [30], and plane structure [31] are calculated as the primitives for registration. Then, using various matching strategies, such as geometric consistency constraints [27], Hough transform [32], search for inliers [33], and non-cooperative games [34], the corresponding features are identified to calculate the transformation between point clouds. The accuracy of registration based on these methods greatly depends on the quality of feature extraction; however, the robustness, generalizability, descriptive capacity, and uniqueness of geometric features may not be sufficient, especially in point clouds with curvature-invariant surfaces.

Instead of feature descriptor calculation and corresponding identification, the four-point congruent set (4PCS)-based registration methods [35] find a transformation between point clouds using constant affine invariance ratios for the distances between pairs of points. These methods are highly efficient with good anti-noise ability [36] and can work well for point cloud sets with small overlaps. Mellado et al. [37] greatly enhanced the 4PCS algorithm by applying a splitting and indexing strategy. Semantic keypoint-based 4PCS (SK-4PCS) [38] combines the advantages of keypoint-based 4PCS (K-4PCS) [39] to reduce unnecessary points and uses the semantics of keypoints to decrease the complexity of the search for corresponding congruent sets. Nevertheless, the coplanar sets in the 4PCS-based algorithm may not take full advantage of the geometric features of planar point clouds.

Another coarse registration scheme is the probabilistic method. These methods are highly robust to outliers, noise, and occlusions. However, because it is highly time-consuming, the utility of probabilistic registration is severely limited. Jian et al. represented the distributions of point clouds as Gaussian mixture models (GMMs), which transform the registration problem into minimizing the statistical discrepancy between two GMMs. The coherent point drift (CPD)-based method [40] uses GMMs to describe the corresponding relationship between two sets of points and considers the registration to be a problem of probability density estimation, which can be solved by the expectation-maximization (EM) algorithm. Gao et al. [41] proposed a novel probabilistic registration method, FilterReg, which uses a Gaussian filter and twist parameterization to achieve robust point cloud registration with fast computational performance.

Recently, deep learning-based methods have been used to solve the problem of 3D point cloud registration. 3DMatch [42] establishes correspondences by learning a local volumetric patch descriptor. Deng et al. [43] presented PPF-FoldNet, which uses a folding-based auto-encoder to learn 3D local descriptors. Aoki et al. [44] considered Point Net as learnable “imaging” and introduced the Lucas and Kanade (LK) algorithm to achieve 3D point cloud registration. The deep closest point [45] method proposes a simple architecture, which addresses key issues in each part of the classical ICP pipeline to obtain the transformation between a pair of point clouds. These deep learning-based methods achieve outstanding performance at small-scale point cloud registration; however, limited by the calculation complexity and the amount of data, they do not apply to large-scale point clouds.

### 2.3. Fine Registration

ICP [5] is the best-known algorithm for fine registration, which alternates between searching point cloud correspondences and finding the optimal solution by the least-squares method to update the alignment. However, owing to the establishment of correspondences by searching the nearest points in the iterative process, the ICP algorithm has low computational efficiency and often stalls at suboptimal local minima. Therefore, several ICP variants have been proposed to solve these problems. To broaden the basin convergence of ICP, Fitzgibbon [6] introduced the Levenberg–Marquardt ICP algorithm that enlarges the range of convergence and improves computational efficiency. Yang et al. [7] proposed a global optimal ICP (Go-ICP) to solve the local minimum problem. However, Go-ICP is sensitive to occlusion and partial overlap and is significantly more time-consuming than ICP. To improve the accuracy and efficiency, point-to-plane [8], point-to-projection [9], and plane-to-plane [10] correspondence error metrics have been used in ICP variants.

Magnusson [46] extended the normal distribution transform (NDT) algorithm to 3D space to realize registration. The main concept of this method is to present a point cloud pair as a set of Gaussian distributions. Subsequently, the Hessian matrix method is used to optimize the probability of the Gaussian distribution of the point cloud pair to realize registration. In contrast to the ICP algorithm, NDT and its variants [47] achieve fine registration without a good initial pose and avoid time-consuming searches for the closest points.

In summary, it is difficult for most extant 3D point cloud registration algorithms to achieve accurate registration for point cloud with curvature-invariant surfaces. In addition, for large-scale point clouds, it is hard work to reduce the processing time while retaining the registration accuracy.

## 3. Proposed Methods

**Figure 1:**
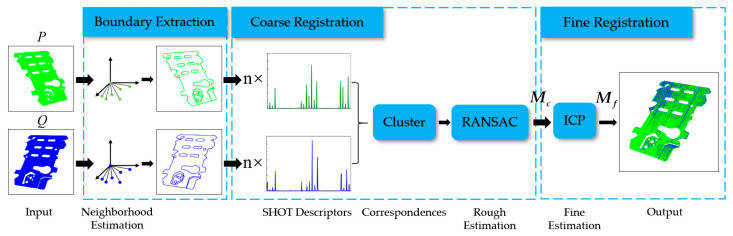
Flowchart of the proposed registration method for a large-scale planar point cloud.

The proposed registration method for a large-scale planar point cloud follows the same framework as the SHOT-ICP algorithm but is optimized hierarchically. Figure 1 shows the flowchart of this method, which involves three steps. The details are presented as follows:

1. Boundary Extraction: Given the input point cloud sets *P* and *Q*, we introduce a novel boundary extraction algorithm to obtain the boundary point clouds efficiently and eliminate the noise caused by the acquisition equipment.
2. Coarse Registration: After boundary extraction, the obtained point clouds are de-scribed by SHOT descriptors. To obtain accurate correspondences, we designed an invalid keypoints elimination method based on the least square (LS) fitting of point clouds in the neighborhood of keypoints. Then, we obtain a cluster of keypoint pairs based on the geometric consistency constraints. Subsequently, the random sample consensus (RANSAC) iteration is used to eliminate further the mismatched keypoints and estimate the approximate transformation ***M_c_***.
3. Fine Registration: Finally, the roughly registered boundary point clouds are combined into the ICP algorithm to obtain the fine transformation ***M_f_***. By adopting two steps of rotation and translation with the parameters ***M_f_***, the input point clouds can be registered accurately.

### 3.1. Optimized Boundary Extraction Algorithm

**Figure 2:**
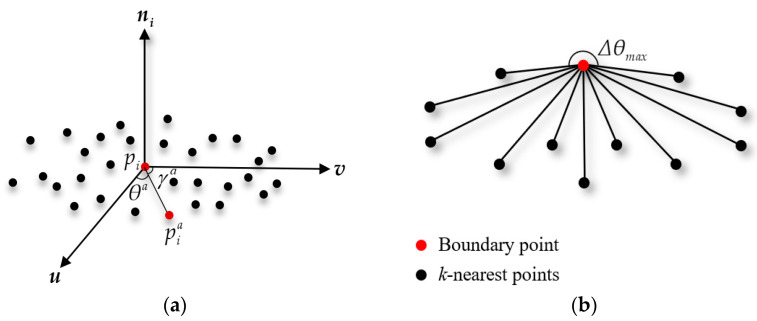
Basic principle of boundary extraction. (**a**) The point-neighborhood evaluation method; (**b**) An illustration of a boundary point.

To achieve more efficient and accurate registration, we characterize the large-scale planar point cloud as its boundary points. The proposed boundary extraction method is a variant algorithm that introduces a hierarchical strategy to improve the computational efficiency and combines the statistical outlier removal filter (SOR) [48] to remove the noise around the boundary. Figure 2 shows the principle of the traditional *k* neighbors angle method-based boundary point cloud extraction method in a point cloud library (PCL).

As shown in Figure 2a, the boundary extraction method is based on the point neighborhood. The neighborhood of each point $p_{i}$ is defined as a set that includes the *k*-nearest points of the center $p_{i}$, where *k* = 30. The covariance matrix ***C*** with dimension 3 × 3 is created to estimate the surface normal and the eigenvectors and eigenvalues are calculated by eigen-decomposition:

(1) $${\bar{p}}_{i}=\frac{1}{k}\sum_{a=1}^{k}p_{i}^{a}$$

(2) $$C=\frac{1}{k}\sum_{i=1}^{k}\left(p_{i}^{a}-\bar{p}\right){\left(p_{i}^{a}-\bar{p}\right)}^{T}$$

(3) $$Cv_{j}=\lambda_{j}v_{j},\;j\in \left\{1,2,3\right\}$$

where $p_{i}^{a}$, a∈(1,k) is the *a*-th *k*-nearest point of $p_{i}$, ${\bar{p}}_{i}$ is the centroid of all *k*-nearest points of $p_{i}$, and $v_{i}$ and $\lambda_{i}$ respectively are the *j*-th eigenvectors and eigenvalues of the covariance matrix ***C***. The eigenvector corresponding to the minimum eigenvalue is identified as the normal vector of the neighborhood. For the normal vectors of all points, the viewpoint position is introduced to solve the problem of ambiguous orientation. Then, based on the normal vector $n_{i}$, the unit orthogonal vectors ***u***, ***v*** are obtained to construct the local coordinate system.

In the local coordinate system of the $p_{i}$, by connecting $p_{i}$ and $p_{i}^{a}$, we can obtain the dot product of $\left(p_{i}-p_{i}^{a}\right)$ with ***u*** and ***v***. Since $p_{i}^{a}$ is probably distributed around the plane $up_{i}v$; there is an approximate formula: $\theta^{a}+\gamma^{a}=\pi /2$. Hence, $\theta^{a}\in \left(-\pi ,\pi \right)$ can be calculated as:

(4) $$\frac{\left(p_{i}-p_{i}^{a}\right)u}{\left(p_{i}-p_{i}^{a}\right)v}=\frac{{\left\|p_{i}-p_{i}^{a}\right\|}_{2}{\left\|u\right\|}_{2}cos\theta^{a}}{{\left\|p_{i}-p_{i}^{a}\right\|}_{2}{\left\|v\right\|}_{2}cos\gamma^{a}}=\frac{cos\theta^{a}}{cos\gamma^{a}}=tan\theta^{a}$$

(5) $$\theta^{a}=arctan2\left[\left(p_{i}-p_{i}^{a}\right)v,\left(p_{i}-p_{i}^{a}\right)u\right]$$

$\theta^{a}$ is calculated for each *k*-nearest point, which is sorted in ascending order to obtain the set, $\left\{\theta_{1},\theta_{2},\theta_{3},\ldots ,\theta_{k}\right\}$. Subsequently, the differences between two adjacent angles are obtained as $\Delta \theta_{t}=\theta_{t+1}-\theta_{t}$ and t∈{1,2,…k−1}, which can be transferred to $\Delta \theta_{t}=2\pi -\theta_{t}+\theta_{1}$, and t=k. If the maximum angle difference is $\Delta \theta_{max}=\underset{1\leq t\leq k}{max}\Delta \theta_{t}$ above a certain threshold π/2, the point $p_{i}$ can be identified as a boundary point cloud, as shown in Figure 2b.

**Figure 3:**
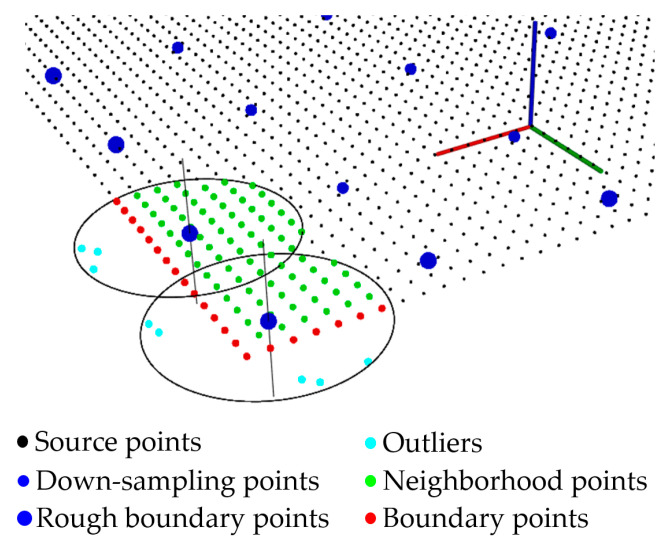
Result of the optimized boundary extraction method. The down-sampling points are first obtained to identify the rough boundary points, and then, we search for the accurate boundary points and eliminate the outliers based on the neighborhood points of the approximate boundary points.

The traditional boundary extraction algorithm can achieve good results, but it is time-consuming if all points are involved in the operation. Furthermore, the original point cloud data must be filtered effectively and denoised, which can enhance the accuracy of subsequent processing. To solve these problems, we propose an improved boundary extraction algorithm, which first obtains approximate boundary points and then identifies the accurate boundary points and removes the outliers through a radius search of the rough boundary points (Figure 3). The working details are presented in Algorithm 1. In this study, the outlier removal factor α is set to 0.5.
**Algorithm 1.** Optimized Boundary Extraction Method**Input:** Source point cloud *S* **Output:** Boundary point cloud *T* without outliers
1*V*←Voxel Down-sampling *S*2**for** each point $p_{i}$ in *V*, **do**3 ${\bar{p}}_{i}=\frac{1}{k}\sum p_{i}^{a}$4 $C=\frac{1}{k}\sum \left(p_{i}^{a}-{\bar{p}}_{i}\right){\left(p_{i}^{a}-{\bar{p}}_{i}\right)}^{T}$5 $Cv_{j}=\lambda_{j}v_{j}$, j∈{1,2,3}6 Boundary identification7 Add boundary points into *R*8**end**9*Z*←radius search for each point in *R*10**for** each point $q_{i}$ in *Z* **do**11 Search for the *k*-nearest points: $q_{i}^{a}$, i∈(1,k)12 $d_{i}=\frac{\sum {\left\|q_{i}-q_{i}^{a}\right\|}_{2}}{k}$13**end**14$\mu \leftarrow \frac{\sum d_{i}}{n}$15$\sigma \leftarrow \sqrt{\frac{\sum d_{i}^{2}-\frac{{\left(\sum d_{i}\right)}^{2}}{n}}{n-1}}$16**for** each point $q_{i}$ in *Z*, **do**17 **if** $d_{i}\geq \mu +\alpha \sigma$ **then**18  **continue**19 **end**20 Boundary identification21 Add boundary points into *T*22**end**


### 3.2. Elimination of Invalid Keypoints

To establish the local correspondences between two boundary point clouds to realize coarse registration, we obtained the keypoints of the boundary by uniform down-sampling and calculated their descriptors. We utilize a robust descriptor capable of reflecting the geometric structure of the boundary point cloud: SHOT, which constructs a normalized histogram by counting the geometric distribution of point clouds in the constructed local reference frame around the keypoints. In particular, the local reference frame uses an isotropic spherical grid with 32 spatial bins that results from two elevation divisions, two radial divisions, and eight azimuth divisions. Each spatial bin can be further divided into 11 sub-bins according to the spatial distribution. Hence, the SHOT descriptor can ultimately be represented as a 352-dimensional vector. The correspondences can be identified as a keypoint pair, the descriptors’ Euclidean distance of which is less than a certain threshold.

**Figure 4:**
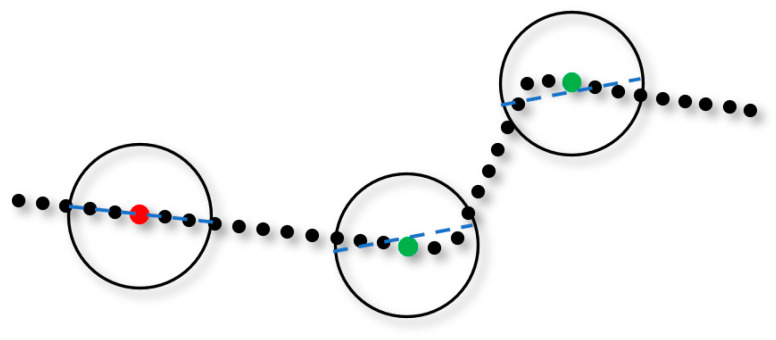
An example of an invalid keypoint elimination method based on LS fitting. The blue dashed lines represent the results of LS fitting in the neighborhood of the keypoints. The green and red dots represent the valid and invalid keypoints, respectively.

Boundary-based SHOT descriptors can overcome the problem of weak uniqueness in a curvature-consistent surface, thus reducing the number of mismatches. However, there are still many non-corresponding keypoint pairs with similar spatial distributions. Therefore, we designed a method to evaluate the neighborhood of each keypoint based on LS fitting to remove invalid keypoints distributed on the linear boundary that may cause mismatches due to their ambiguous descriptors. As shown in Figure 4, point clouds in the neighborhood of each keypoint are fitted as an LS straight line, and the average distance from all point clouds to the LS line is calculated and is used to evaluate the descriptiveness of the descriptor of the keypoint. If the average distance is less than a certain threshold, the keypoint is identified as invalid.

The target LS line can be represented by a unit vector, D and the centroid in the neighborhood ***g***, yielding:

(6) l(ε)=εD+g

The objective function of LS fitting can be defined as:

(7) $$f=\sum_{j=1}^{n}\left[{\left\|G_{j}\right\|}_{2}^{2}-{\left(G_{j}D\right)}^{2}\right]$$

where *n* is the number of neighbor points and $G_{j}$ is the vector that connects the centroid with every other point in the neighborhood. Owing to the constraint $D^{T}D=1$, ${\left\|G_{j}\right\|}_{2}^{2}$ can be written as $D^{T}\left(G_{j}^{T}G_{j}\right)D$, and Formula (6) can be transformed to:

(8) $$f=D^{T}\sum_{j=1}^{n}\left[\left(G_{j}^{T}G_{j}\right)E-G_{j}G_{j}^{T}\right]D,\;\mathrm{and}\;H=\sum_{j=1}^{n}\left[\left(G_{j}^{T}G_{j}\right)E-G_{j}G_{j}^{T}\right]$$

where is ***E*** a three-dimensional unit matrix. To find the minimum, the key is to obtain the eigenvalues and eigenvectors of ***H***. The details of the invalid key point elimination method are given in Algorithm 2 from which we can obtain the coarse correspondences. The elimination threshold, ζ=0.2γ, and the correspondence identification threshold, ψ=3γ are utilized in this study. γ is the resolution of the input point cloud, which can be calculated as:

(9) $$\gamma =\frac{\sum_{i=1}^{m}{\left\|c_{i}-c_{i}^{\prime }\right\|}_{2}}{m}$$

where $c_{i}$ is the *i*-th point in the point cloud, $c_{i}^{\prime }$ is its nearest neighbor point, and *m* is the number of points in the input point cloud.
**Algorithm 2.** Invalid Keypoints Elimination Method**Input:**$K^{\prime }$, $K^{\prime \prime }$, $f^{\prime }$, $f^{\prime \prime }$: Keypoints sets and their corresponding SHOT descriptors sets of input point cloud pair
 **Output:** Coarse correspondences *C*
1**for** each point $p_{i}$ in $K^{\prime }$ **do**2 $H\leftarrow \sum \left[\left(G_{j}^{T}G_{j}\right)E-G_{j}G_{j}^{T}\right]$3 $Hi_{j}=\lambda_{j}i_{j}$, j∈{1,2,3}4 Sorting the eigenvalues: $\lambda_{1}\leq \lambda_{2}\leq \lambda_{3}$, and the corresponding eigenvectors are $i_{1}$, $i_{2}$, $i_{3}$5 **if** $\frac{\lambda_{1}}{n}>\zeta$ **then**6  **continue**7 **end**8 $d_{c}\leftarrow min{\left\|f_{i}^{\prime }-f_{j}^{\prime \prime }\right\|}_{2}$9 **if** $d_{c}<\gamma$ **then**10  Add $\left(K_{i}^{\prime },K_{j}^{\prime \prime }\right)$ into *C*;11 **end**12**end**


### 3.3. Correspondence Clustering

Given the coarse correspondences, point cloud registration can be achieved by the RANSAC algorithm. RANSAC iteratively and randomly samples a subset to generate the hypothesis transformation by the singular value decomposition (SVD) method and then tests all the remaining correspondences to detect the best transformation, which is defined as the transformation with the highest percentage of inliers. 

The time complexity in the RANSAC iteration depends on the number of input correspondences and the inlier ratio. Therefore, we cluster correspondences based on geometric consistency constraints before inputting them to the RANSAC framework to improve the convergence rate. We iteratively consider the correspondence of the descriptor with the smallest Euclidean distance as the center of a hypothesis cluster and then expand the cluster with the remaining correspondences of which the geometric consistency evaluation values to the cluster center are less than the threshold φ=1. Subsequently, we identify a valid hypothesis cluster for which the membership exceeds a certain threshold, η=50. The working details of the correspondence clustering are described in Algorithm 3.
**Algorithm 3.** Correspondence Clustering**Input:** Coarse correspondences *C* **Output:** Fine correspondences *M*
1Sort *C* in ascending order according to the Euclidean distance between two descriptors of each matching pair to attain a new set of correspondences: $C^{\prime }=\left\{\left(u_{i},v_{i}\right)|i=1,2,3\ldots ,n\right\}$ and $c_{i}=\left(u_{i},v_{i}\right)$2**for** each correspondence $c_{i}$ in $C^{\prime }$ **do**3 **if** $c_{i}$ is unavailable **then**4   **continue**5 **end**6 Assume a cluster $C_{a}$, and then add $c_{i}$ into $C_{a}$7 **for** each correspondence $c_{j}$ in $C^{\prime }$ **do**8  **If** $c_{j}$ is unavailable or j=i, **then**9   **continue**10  **end**11  $\Delta d_{ij}\leftarrow {\left\|u_{i}-u_{j}\right\|}_{2}-{\left\|v_{i}-v_{j}\right\|}_{2}$12  **If** $\Delta d_{ij}\leq \varphi$, **then**13   Add $c_{j}$ into $C_{a}$14  **end**15 **end**16 **if** $C_{a}.size>\eta$, **then**17  Add $C_{a}$ into *L*18  Set $c_{i}$ in $C_{a}$ unavailable19 **end**20**end**21Sort *L* in ascending order according to the number of correspondences of the cluster to attain a set $L^{\prime }=\left\{C_{k}|k=1,2,3,\ldots ,m\right\}$22$M\leftarrow C_{m}$


### 3.4. Registration and Flatness Measurement

According to the fine correspondences set, the coarse transformation ***M_c_*** between point cloud pairs can be calculated using the RANSAC and SVD algorithms. Subsequently, fine registration is completed by submitting a coarse registered boundary point cloud into the ICP framework, which greatly reduces the computational complexity and retains precision. The final transformation matrix ***M_f_*** can be expressed as:

(10) $$M_{f}=\left(\begin{array}{cc} R & T\\ 0 & 1 \end{array}\right)$$

where ***R*** is the rotation matrix with 3 × 3 dimensions and ***T*** is the translation vector with 3 × 1 dimensions.

**Figure 5:**
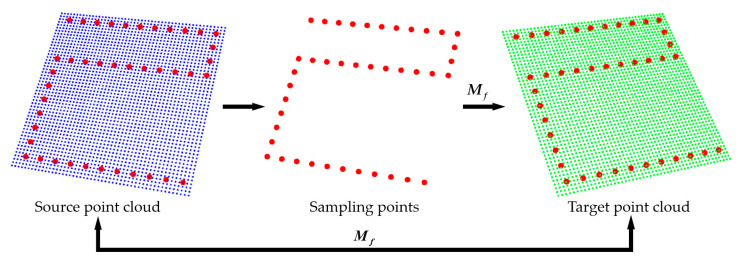
Illustration of the mapping of flatness sampling points. Sampling points (**red** dots) from the coordinate system of the source point cloud (**blue** one) to the coordinate system of the target point cloud (**green** one).

To ensure repeatability in flatness measurements, the consistency of flatness sampling locations must be ensured. As shown in Figure 5, we first set a point cloud with the standard position as the source point cloud and set the sampling points in its coordinate system. Then, the point cloud of the object to be measured is used as the target point cloud, and the transformation matrix ***M_f_*** between the two point clouds is obtained. Finally, we utilize the transformation matrix ***M_f_*** to map the standard sampling points to the coordinate system of the target point cloud and search for the nearest points of the converted sampling points as valid data points to achieve the flatness measurement. The mapping of points can be expressed as:

(11) $${\left(x_{i}^{\prime },y_{i}^{\prime },z_{i}^{\prime },1\right)}^{T}=\left(\begin{array}{cc} R & T\\ 0 & 1 \end{array}\right){\left(x_{i},y_{i},z_{i},1\right)}^{T}$$

where $\left(x_{i},y_{i},z_{i}\right)$ is the standard sampling point, and $\left(x_{i}^{\prime },y_{i}^{\prime },z_{i}^{\prime }\right)$ is the converted sampling point.

## 4. Experimental Results and Discussion

**Figure 6:**
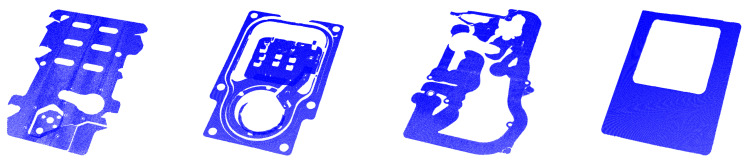
Display of point clouds for registration with different parameters, the point clouds and their average resolutions from left to right are $M_{1}:\gamma_{1}=0.2782$, $M_{2}:\gamma_{2}=0.0815$, $M_{3}:\gamma_{3}=0.2496$, and $M_{4}:\gamma_{4}=0.0754$, respectively.

To verify the efficiency of the proposed method, we first evaluated our optimized boundary extraction algorithm based on different large-scale planar point clouds, which were acquired with a profilometer. Then, the proposed mismatched elimination method was evaluated on the same dataset. Third, the performance and computational efficiency of our registration algorithm were compared with those of other methods. Finally, the proposed registration method is applied to realize laser profilometer-based flatness measurements. As shown in Figure 6, the point cloud dataset includes four point clouds, *M*_1_, *M*_2_, *M*_3_, and *M*_4_, which were scanned from the four models using the LMI Gocator 2350 laser profilometer, were used in our experiments. The experiments were conducted based on an open-source PCL. All experiments in this study were run on a computer equipped with an AMD Ryzen 7 4800H, 2.9 GHz CPU, and 16.0 GB of memory.

### 4.1. Boundary Extraction

For the first experiment, we validate the boundary extraction algorithm proposed in this paper by comparing it with the SOR-boundary extraction (BE) algorithm in PCL. The SOR-BE makes all points involved in the calculation, which significantly increases the processing time. In contrast, our approach first obtains a rough boundary to select the region of interest (ROI); then, it contrapuntally removes outliers and extracts boundary points in the ROI to reduce computational complexity.

**Figure 7:**
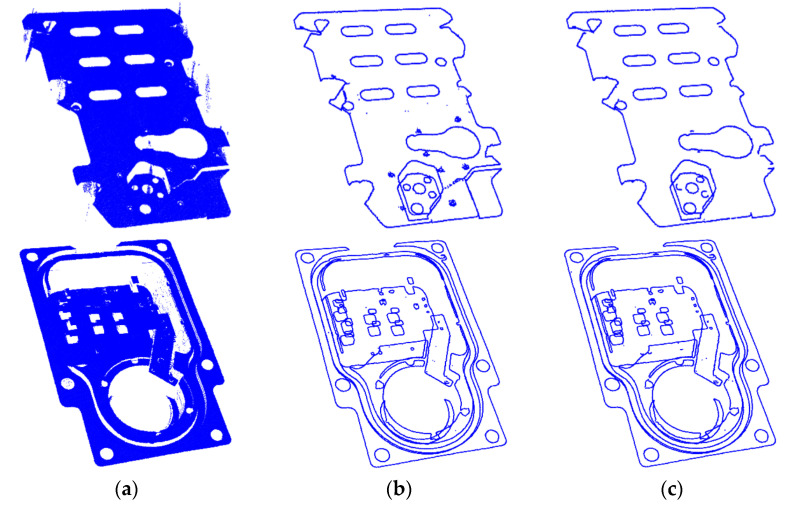
Boundary extraction result from different point clouds based on two methods. (**a**) Input point clouds; (**b**) Extraction results with SOR-BE method; (**c**) Extraction results with our method.

Table 1 shows the quantitative results of the computational complexity of the two methods. Since the proposed algorithm reduces the number of points in the calculation by at least 61.7%, the processing time is reduced by more than 55.7%. In particular, for point clouds with a simple boundary composition, the acceleration effect of our approach is more obvious, and the processing time can be reduced by 75.8%. In addition, the number of boundary points obtained by our method is approximately the same as that of the SOR-BE algorithm. Figure 7 shows the comparison results of the boundary extraction for point clouds in *M*_1_ and *M*_2_ using the two methods. Owing to the use of voxel down-sampling and ROI selection, our algorithm has better anti-noise performance but suffers from reduced extraction capability for tiny boundaries.

### 4.2. Mismatches Elimination

**Figure 8:**
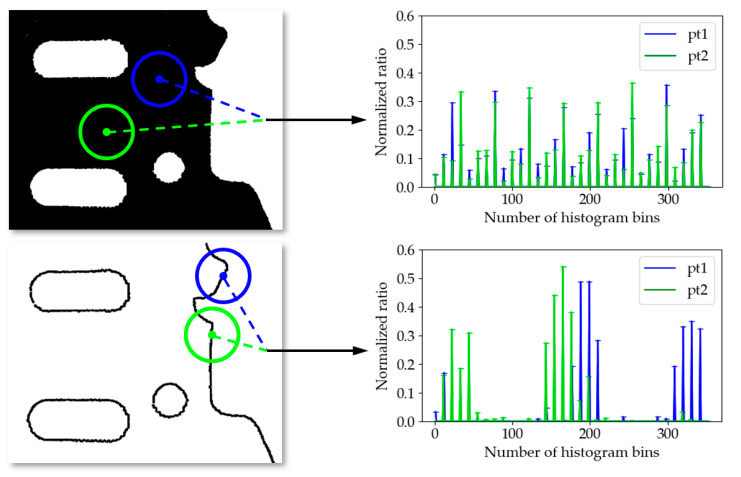
Comparison results of the uniqueness of descriptors between a planar point cloud and its corresponding boundary point cloud. For each point cloud, we select two sampling points and calculate their SHOT descriptors. The histograms show the differences between the two descriptors.

Feature matches based on boundary point clouds can overcome the problem of mismatch caused by the weak uniqueness of descriptors in planar point clouds. For comparison, we calculated the SHOT feature descriptor before and after the boundary extraction of the *M*_1_ point cloud, and the comparison results are shown in Figure 8. Since most of the space composition of the planar point cloud is almost identical, the resulting feature descriptors tend to be similar, and mismatched pairs are prone to occur. In contrast, the feature descriptors for the point cloud after boundary extraction are unique, which results in more correspondences that are accurate.

**Figure 9:**
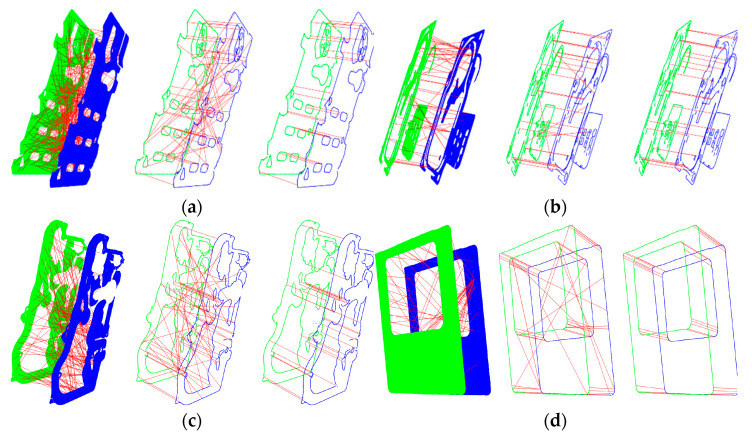
Comparison results of accuracy of correspondences under several conditions for different point clouds. (**a**) Comparison results under different conditions of *M*_1_ point clouds; (**b**) *M*_2_; (**c**) *M*_3_; (**d**) *M*_4_.

Correspondences can be obtained by calculating the Euclidean distances between the descriptors. The proposed invalid keypoints elimination and correspondence clustering algorithm can improve the accuracy of matching point pairs. We experimented on four point clouds and compared the accuracy among original correspondences, boundary-based correspondences, and correspondences after using our identification algorithm. Figure 9 displays the comparison result and, for a more intuitive presentation, we rotate a pair of point clouds parallel to each other according to the rotation matrix between them and display their correspondences. In this case, a higher proportion of parallel lines between two point clouds means a higher proportion of correct correspondences.

As shown in Figure 9, there are a large number of mismatched pairs in planar point clouds, which may lead to a waste of computation and a decrease in the accuracy of point cloud registration. In this case, we extract the boundary of the point cloud and recompute the descriptors to match the keypoints, because the number of invalid descriptors decreases while the descriptive capacity of descriptors is enhanced, and the ratio of mismatches decreases significantly. Subsequently, the proposed invalid keypoint elimination and correspondence clustering algorithm are further used to identify the correspondences. Table 2 shows the quantification result of the inlier ratio of correspondences, where we use the remaining correspondences after RANSAC iteration as the ground truth. For all the different models, mismatches in the original point clouds are more numerous than those in the boundary point clouds; however, for textured point clouds such as *M_2_*, boundary extraction slightly improves the descriptive capacity of the descriptors. In addition, the proposed correspondences identification algorithm can maintain the inlier ratio of correspondences above 93.6%, which provides a good input dataset for the RANSAC algorithm to achieve efficient coarse registration.

### 4.3. Point Cloud Registration

In this experiment, we evaluated the registration performance of the proposed algorithm. For comparison, several coarse-to-fine strategy methods, such as SHOT-ICP (SICP), Super4PCS-ICP (SPICP), and another global registration method, NDT, were applied in our experiment. We used point clouds in the dataset (*M*_1_, *M*_2_, *M*_3_, *M*_4_) for registration, and each type of point cloud in the dataset was obtained from different views by using the laser profilometer. The root-mean-square error (RMSE), which calculates the Euclidean distance between transformed input sets, was adopted to evaluate the accuracy of the point cloud registration.

(12) $$\mathrm{RMSE}=\sqrt{\frac{1}{N}\sum_{k=1}^{N}{\left\|R_{f}p_{k}+T_{f}-q_{k}\right\|}_{2}^{2}}$$

where $p_{k}$ and $q_{k}$ are the points in the input point cloud sets, and ***R_f_*** and ***T_f_*** are the rotation matrix and translation vector in the final transformation matrix ***M_f_***.

The processing time and registration accuracy of these algorithms were tested first. We tested all algorithms with the same iteration-stopping criteria; either the number of iterations exceeded 50 or the difference between adjacent transformations was less than the threshold ε. Since the setting of the threshold ε is subject to the point cloud density, we set ε=0.0005γ, where γ is the resolution of the input point cloud. To average the variance caused by point cloud acquisition devices and enhance the reliability of the experiment, we selected five pairs of samples from each type of point cloud and ran the algorithm 20 times for each pair to report the average values of the results.

The experimental results of the four methods are listed in Table 3. It is apparent that the proposed algorithm performs better in the registration of large-scale planar point clouds than the other three algorithms. Compared with other methods, the proposed method saves at least 39.93% of the average runtime in the coarse registration stage and reduces the average total runtime by at least 34.42%. The proposed method makes two major contributions that significantly reduce registration runtime. In the coarse registration stage, the proposed mismatches-elimination method effectively increases the ratio of inlier correspondences and therefore accelerates the convergence speed of the RANSAC algorithm. Conversely, in the fine registration stage, because the boundary-based ICP greatly reduces the number of points in the iteration, the computational efficiency is significantly improved.

**Figure 10:**
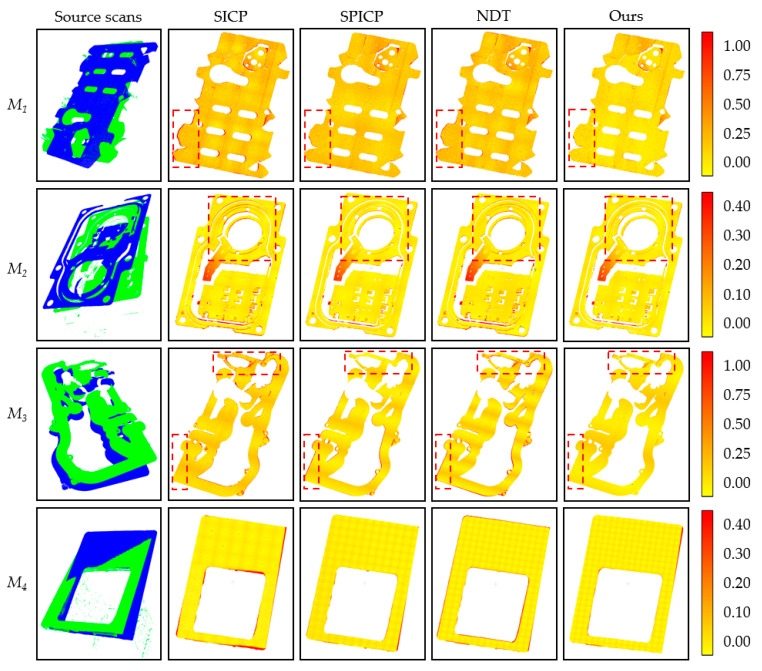
Registration results of the four measured models with the four registration methods (SICP, SPICP, NDT, and Ours). The first column shows the two source scans for each model. The registration error for each model is measured by the change of color according to the color bar, and the measurement unit is mm.

The SPICP algorithm is more effective than other methods for registration accuracy on textured point clouds, but our method exhibits a smaller error for textureless point clouds. In the SICP algorithm, registration accuracy is limited to the uniqueness of the SHOT descriptors. As for the SPICP algorithm, the coplanar sets cannot take full advantage of the geometry of the planar point cloud, which affects the accuracy of registration. In contrast, our method can overcome these problems and achieve accurate point cloud registration based on boundary information. Figure 10 shows the visualization results of registration using these four algorithms, which indicates that in most cases, the proposed method performs better with less registration error than the other three methods.

**Figure 11:**
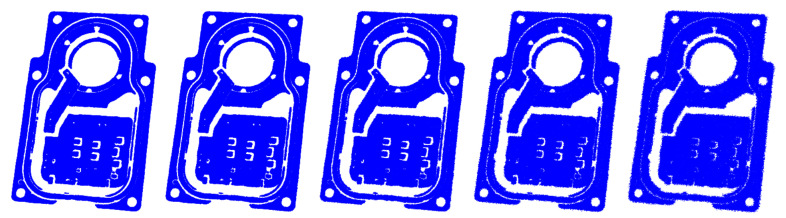
Gaussian noise is added to the point cloud in the *M_2_* point cloud sample; the standard deviations from left to right are 0.1, 0.2, 0.3, 0.4, and 0.5.

To evaluate further the algorithms, we tested the stability of the proposed method for Gaussian noise perturbation. The scanned data usually contain noise points, resulting in multiple errors. In addition, the noise points are attached to the point cloud rather than being free, which makes it difficult to filter out completely and affects the registration result. Hence, the anti-noise ability of the algorithm is significant. Gaussian noise was added to a certain sample in each type of point cloud, and the standard deviations were 0.1, 0.2, 0.3, 0.4, and 0.5. For each sample, we ran each algorithm 100 times to report the average registration errors to eliminate the random noise effect. Figure 11 shows the results of adding different levels of Gaussian noise to the *M*_2_ point cloud sample.

**Figure 12:**
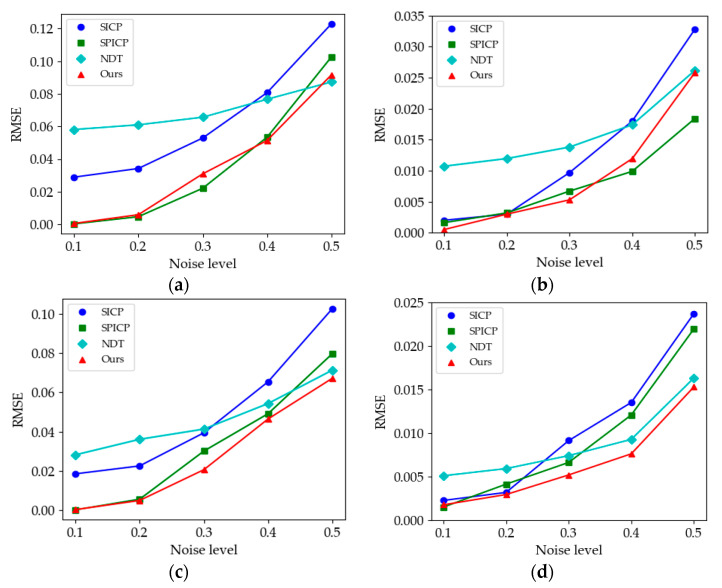
Comparison of average RMSE of the four algorithms at different noise levels for different point clouds. (**a**) Comparison results of *M*_1_ point cloud; (**b**) *M*_2_; (**c**) *M*_3_; (**d**) *M*_4_.

Figure 12 shows the results of the anti-noise experiment. We used the average RMSE to evaluate the different algorithms’ robustness to noise. Figure 12a–d displays similar registration error trends; SPICP and our method are more robust to noise, especially in the case of low noise interference. In contrast, because the NDT algorithm achieves point cloud registration based on optimizing the probability distribution rather than searching for the nearest point to iterate, it is less affected by the increased noise level but has worse performance at low noise interference than the other algorithms. In addition, the descriptive capacity of descriptors in point clouds with a curvature-consistent surface is susceptible to noise, resulting in the low anti-noise ability of the SICP. By comparing Figure 12a–d, the RMSEs of the same algorithm on different models are not the same. This may be caused by different model resolutions, structures, or surface features, but overall, our method achieves the best performance among all models.

**Figure 13:**
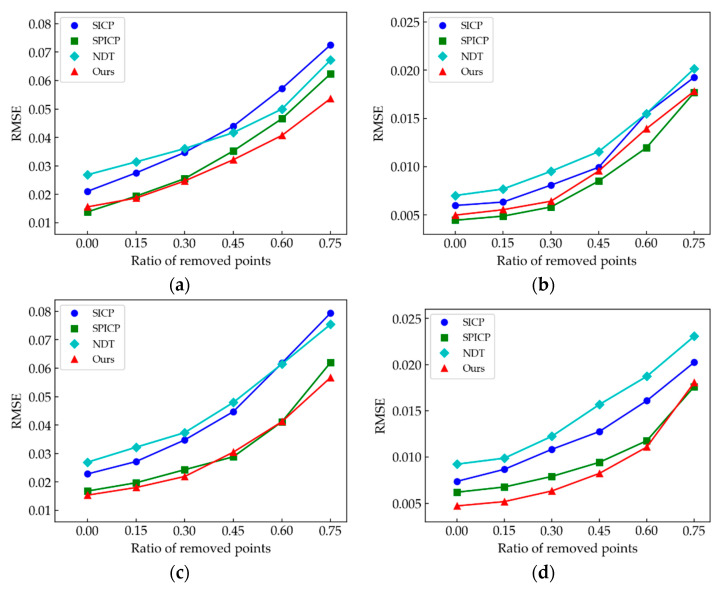
Evaluation of the impact of uniform down-sampling with different sampling densities on the four algorithms. (**a**) Comparison results of *M*_1_ point cloud; (**b**) *M*_2_; (**c**) *M*_3_; (**d**) *M*_4_.

**Figure 14:**
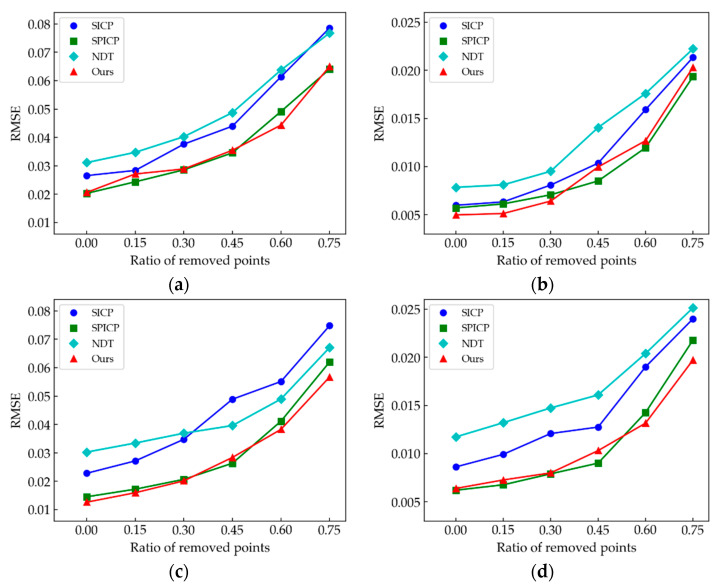
Evaluation of the impact of Poisson disk down-sampling with different sampling densities on the four algorithms. (**a**) Comparison results of *M_1_* point cloud; (**b**) *M*_2_; (**c**) *M*_3_; (**d**) *M*_4_.

The algorithms’ performance for registering point clouds with different sampling structures and densities was also tested. We sampled the point cloud models in dataset (*M*_1_, *M*_2_, *M*_3_, *M*_4_) to get the synthetic data. Uniform and Poisson disk sampling structures were utilized to sample the model point clouds. To perform a controlled evaluation, we set the ratio of the removed points from 0 to 0.75 to represent different sampling densities for both sampling modes. Similarly, RMSE between registered synthetic data and a model point cloud was used to reflect the stability of the registration algorithm for different sampling structures and densities. To average the variances caused by point cloud down-sampling, we ran 20 times for each pair of point clouds and reported the average values of results. Figure 13 and Figure 14 show the results of the registration errors of the four algorithms under different sampling perturbation.

As shown in Figure 13 and Figure 14, with the decrease of the remaining points, RMSEs all show an upward trend, but for different sampling structures, the registration errors are different. Since the Poisson disk sampling generates new random points, and the uniform sampling removes a certain number of points from the origin data, the former changes the structure of the origin point cloud more significantly, which causes larger RMSE. In general, the registration errors of NDT and SICP algorithms are larger under different sampling structures and densities, while our method and SPICP have higher accuracy. Since the SHOT descriptor is normalized to sum up to 1, SHOT-based algorithms achieve robustness to variations of the point densities. In addition, our method further enhances the descriptive capacity of the descriptor, thus achieving more accurate point cloud registration.

### 4.4. Application to Flatness Measurement

The proposed algorithm was applied to flatness measurements based on a laser profilometer to verify the effect of point cloud registration accuracy on the measurement results. We tested five different types of objects corresponding to *M*_1_, and for each type, we constantly changed its pose in the process to obtain 20 results to report the average value of the results and their standard deviation. For a more significant comparison, as a control, we selected the SICP, the performance of which differed most from our registration methods. Furthermore, we used the measurement from a ZEISS CONTURA 9/16/8 RDS coordinate measuring machine (CMM) as a reference, the sampling points of which are set to be the same as the standard sampling points in the point cloud.

Table 4 shows the average and standard deviations obtained by the two methods. The results obtained by these algorithms were approximately the same as the reference values. However, owing to the higher accuracy of our registration method, the reproducibility standard deviation of the resulting measurements was smaller and could be controlled below 0.0023 mm. In addition, because 200 sampling points were selected in this experiment, the processing time required by the CMM was more than 20 min, whereas the method based on our algorithm combined with the laser profilometer takes less than 20 s, which is more suitable for batch testing.

## 5. Conclusions and Future Work

This paper presents an accurate, efficient, and stable algorithm for large-scale planar point cloud registration. In this approach, coarse-to-fine registration is achieved based on the boundary information. Our improved boundary extraction method filters out the outliers and obtains accurate boundary points after an approximate location. In addition, we introduce an LS-based invalid keypoints elimination method and cluster the correspondences based on geometric consistency constraints to improve the speed and accuracy of registration. The proposed method was tested on four types of large-scale planar point clouds. The experimental results show that our method is both efficient and fast and that it accurately determines the optimal transformation under different levels of noise. In addition, our method successfully overcomes the problems raised in the introduction and can be widely applied to flatness measurement or other applications requiring point cloud registration.

However, the proposed algorithm has certain limitations. The registration accuracy of the method in this study is largely dependent on the accuracy of the boundary extraction result, while it requires experience in selecting optimal down-sampling voxel sizes and search radii in different environments. In addition, the descriptive capacity of the descriptors also affected the results. Future work will include a progressive strategy for boundary extraction and a more efficient descriptor for the boundary point cloud.

## Figures and Tables

**Table 1:** Comparison between boundary extraction results of two methods.

Point Cloud	Number of Original Points	Method	Number of Involved Points	Number of Boundary Points	Processing Time (ms)
*M_1_*	356,522	SOR-BE	356,522	10,140	2235
		Ours	56,038	9688	763
*M_2_*	306,724	SOR-BE	306,724	20,402	1937
		Ours	117,345	19,783	859
*M_3_*	283,972	SOR-BE	283,972	10,829	1864
		Ours	51,787	10,301	597
*M_4_*	340,065	SOR-BE	340,065	4762	2095
		Ours	36,516	4778	507

**Table 2:** Quantification result of the inlier ratio of correspondences under several conditions for different point clouds.

Point Cloud	*M*_1_	*M*_2_	*M*_3_	*M*_4_
Original	7.868%	10.694%	69.716%	13.384%
Boundary	31.462%	45.695%	75.432%	38.747%
Identified	97.191%	100%	98.701%	93.608%

**Table 3:** Performance comparison of different algorithms in coarse and fine registration stages.

Point Cloud	Number of Points	Algorithm	Coarse Registration		Fine Registration	
			Time*_c_* (ms)	RMSE*_c_* (mm)	Time*_f_* (ms)	RMSE*_f_* (mm)
*M*_1_	355,762	SICP	6532	0.4586	21,541	0.0921
		SPICP	3681	0.1746	17,340	0.0561
		NDT	—	—	6947	0.0736
		Ours	2102	0.0821	2603	0.0490
*M*_2_	314,200	SICP	6389	0.0423	19,571	0.0173
		SPICP	5032	0.0296	17,143	0.0153
		NDT	—	—	4526	0.0221
		Ours	2141	0.0311	2968	0.0164
*M*_3_	291,469	SICP	5374	0.2314	16,872	0.0764
		SPICP	4509	0.1689	15,868	0.0433
		NDT	—	—	4698	0.0872
		Ours	1820	0.1052	2327	0.0422
*M*_4_	355,139	SICP	5126	0.1199	18,963	0.0413
		SPICP	3291	0.0456	15,213	0.0213
		NDT	—	—	4971	0.0523
		Ours	1422	0.0350	1650	0.0187

**Table 4:** Average, standard deviation, and the reference value of flatness measurement by the two methods.

	Reference	Average (mm)		Standard Deviation (mm)	
		SICP	Ours	SICP	Ours
1	0.1034	0.0977	0.0986	0.0036	0.0019
2	0.1655	0.1338	0.1395	0.0034	0.0021
3	0.1039	0.0945	0.0938	0.0041	0.0023
4	0.0943	0.1071	0.1082	0.0035	0.0020
5	0.1565	0.1295	0.1336	0.0038	0.0012
